# Molecular mechanism for transcriptional regulation of the parathyroid hormone gene by Epiprofin

**DOI:** 10.1111/febs.70085

**Published:** 2025-03-31

**Authors:** Takashi Nakamura, Hannah M. Nakamura, Yasumasa Iwasaki, Motomi Enomoto‐Iwamoto, Noriaki Nakashima, Satoshi Fukumoto, Maurizio Pacifici, Masahiro Iwamoto, Minoru Wakamori

**Affiliations:** ^1^ Division of Molecular Pharmacology & Cell Biophysics, Department of Disease Management Dentistry Tohoku University Graduate School of Dentistry Sendai Japan; ^2^ Division of Nephrology and Endocrinology Tohoku Medical and Pharmaceutical University Sendai Japan; ^3^ Department of Endocrinology, Metabolism and Nephrology, Kochi Medical School Kochi University Japan; ^4^ Department of Orthopaedics University of Maryland School of Medicine Baltimore MD USA; ^5^ Department of Breast Cancer and Endocrine Surgery Tohoku University Hospital Sendai Japan; ^6^ Division of Pediatric Dentistry Kyushu University Graduate School of Dentistry Fukuoka Japan; ^7^ Division of Orthopedic Surgery, Department of Surgery Children's Hospital of Philadelphia PA USA

**Keywords:** calcium homeostasis, PTH, chronic kidney disease, Epiprofin/Sp6, tooth development

## Abstract

Epiprofin (Epfn), an Sp/KLF family transcription factor that regulates cell proliferation and determines cell fates, is essential for normal skin, hair follicle, and tooth development. We found that *Epfn* was expressed in parathyroid glands, and *Epfn‐*knockout mice displayed elevated serum parathyroid hormone (PTH) concentrations, decreased bone volume, and intracranial ectopic calcification. To investigate the role of Epfn in the regulation of *PTH* expression, parathyroid gland explant and parathyroid cell line culture methods were used. *Epfn* expression was found to be upregulated in response to an increase in extracellular calcium concentration, whereas *PTH* expression was downregulated, thus demonstrating an inverse correlation. Forced expression of *Epfn* inhibited *PTH* gene expression and *PTH* promoter reporter activity in parathyroid cells. In addition, with a high extracellular calcium concentration, *Epfn* silencing in cultured parathyroid glands failed to block *PTH* gene expression. ChIP‐qPCR analysis also revealed Epfn binding in the proximal region of the *PTH* promoter, which was accelerated in the presence of a high concentration of calcium ions. The results from our *in vitro* and *ex vivo* analyses suggest that Epfn is a newly identified negative regulator of *PTH* transcription by regulating the proximal *PTH* promoter. Furthermore, the expression of *Epfn* was significantly reduced in parathyroid adenomas of primary hyperparathyroidism patients. The identification of Epfn as a potential therapeutic target for the control of PTH production in hyperparathyroidism patients opens new avenues for targeted treatment approaches.

## Graphical abstract

The secretion of parathyroid hormone (PTH) from the chief cells in the parathyroid glands is regulated by the concentration of extracellular calcium. Epiprofin (*Epfn*) expression is induced by NFATc2 in response to elevated serum calcium, and Epfn negatively regulates PTH production. Furthermore, forced expression of *Epfn* suppresses PTH production, irrespective  of serum calcium concentration. Epfn is therefore a promising therapeutic target for the regulation of PTH production in patients with primary and secondary hyperparathyroidism.

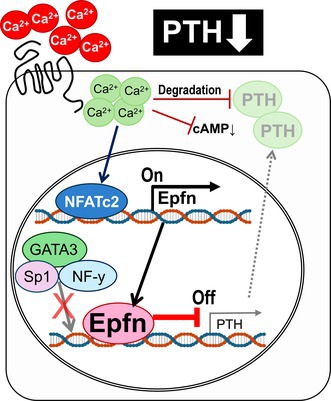

**Abbreviations**
cAMP: cyclic adenosine monophosphate
CaSR: calcium‐sensing receptor
ChIP: chromatin immunoprecipitation
CKD: chronic kidney disease
Epfn: Epiprofin
Epfn KO: Epiprofin knockout
KLF: Krüppel‐like factor
microCT: micro computed tomography
PHPT: primary hyperparathyroidism
PTg: parathyroid gland
PTH: parathyroid hormone
qPCR: quantitative polymerase chain reaction
RT: reverse transcription

## Introduction

Epiprofin (Epfn) is a zinc finger transcription factor that is a member of the Sp family [1]. There are nine Sp family members in mammals (Sp1–Sp9), with Epfn corresponding to Sp6. Each member possesses three C2H2 type zinc finger motifs at the C terminus and binds to GGGCGG motifs or related GC‐rich sequences, and also shares a high degree of structural conservation even outside the zinc‐finger domain [2, 3]. In addition, Epfn also binds the consensus nine nucleotide binding DNA motif CTg/aTAATTA [4]. Sp family transcription factors regulate gene expression either positively or negatively, and influence diverse biological processes including cell proliferation, cell cycle progression, and apoptosis as well as tumorigenesis [5, 6, 7]. Although Sp1 to Sp4 are ubiquitously expressed, Sp5 to Sp9 are expressed in a tissue‐ and developmental stage‐specific manner [8]. For example, Sp7 is predominantly expressed in bone and plays an indispensable role in bone development [9], while *Epfn* (Sp6) is expressed preferentially in developing epithelial cells, such as teeth and skin. Consistent with its expression profile, mice lacking *Epfn* display substantial defects in tooth, skin, and hair follicle development [10, 11, 12, 13, 14]. Furthermore, *Epfn* knockout (KO) mice exhibit thinning of cortical bones and retardation of longitudinal bone growth. *Epfn* expression is barely detectable in bone, indicating that it plays an additional systemic role in the regulation of bone metabolism. Notably, it has been reported that the proximal promoter region of the parathyroid hormone (PTH) gene contains a putative Sp binding site [15, 16], suggesting that Epfn may regulate the gene expression of *PTH*, one of the major regulatory hormones in bone metabolism [17].

PTH regulates calcium homeostasis, acting on bones, kidneys, and indirectly through generation of calcitriol on the intestines [17]. Secretion of PTH from the parathyroid glands (PTgs) is regulated by the concentration of extracellular calcium through interaction with membrane‐bound calcium‐sensing receptors (CaSRs) [18]. When the extracellular calcium level is low, a decrease in parathyroid CaSR activity leads to an increase in PTH secretion; in the short term by release of PTH from PTg cells and in the longer term by *de novo* synthesis of PTH [19]. Mechanisms controlling PTH synthesis include the *PTH* mRNA transcription and stabilization, as well as cell proliferation of chief cells that synthesize PTH [20, 21, 22]. Several transcription factors have been identified as regulators of the *PTH* transcription. The *PTH* gene transcription is activated by Sp1, Sp3, or nuclear factor Y (NF‐y), which are directly binding in the proximal region of the *PTH* gene [15, 23].

Hyperparathyroidism, that is, excess production of PTH, is caused by a parathyroid tumor (primary hyperparathyroidism, PHPT), chronic kidney disease (CKD), or vitamin D deficiency (secondary hyperparathyroidism) [24]. PHPT is associated with hypercalcemia and increased bone turnover, which can lead to reduced bone mass [17, 25]. Elevated PTH also affects the cardiovascular and neuromuscular systems [26]. There is an urgent need for the development of new therapeutic drugs and regimens that reduce PTH production, as the current primary approach is PTg surgery.

Unexpectedly, *Epfn* KO mice displayed a phenotype with characteristics of hyperparathyroidism. This study focused on the role of Epfn in the regulation of *PTH* transcription *in vivo* and *in vitro*, and the findings demonstrated a link between the reduced Epfn level and the overproduction of PTH in parathyroid adenomas.

## Results

### Epfn expression in PTgs

**Fig. 1:**
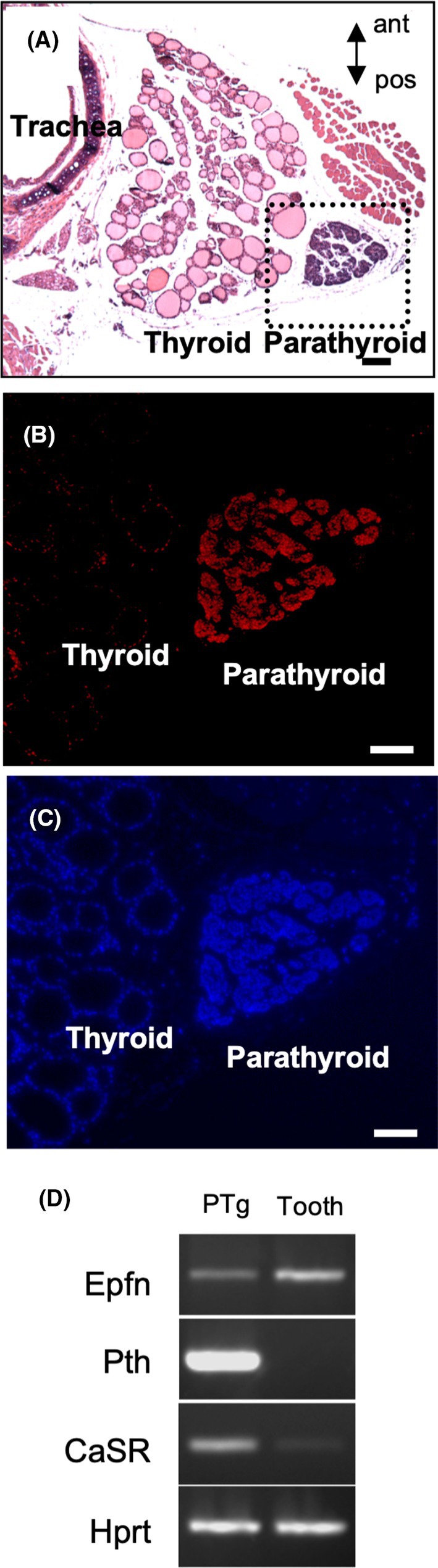
Expression of Epfn in parathyroid glands. (A) Transverse section of 1‐month‐old mouse neck region at the parathyroid gland level was prepared and stained with Hematoxylin–eosin (HE). (B, C) Immunofluorescence analysis of Epfn expression using anti‐Epfn antibody and DAPI. Bar in A indicates 200 μm, and in B and C indicates 100 μm. (D) Gene expression of *Epfn*, *PTH*, and *CaSR* in parathyroid glands (PTg) and tooth buds (Tooth). PCR primer information is presented in Table S3. Immunohistochemistry and RT‐PCR analysis were performed using three independent replicates.

Epfn‐expressing organs involved in mineral metabolism were analyzed for screening purposes using the Protein Atlas (https://www.proteinatlas.org/ENSG00000189120‐SP6/tissue) and the NCBI Unigene database (Hs.253603, Mm 156 282) (Table S1). A high level of expression of Epfn was found in PTgs (Hs.253603), while no positively expressed sequence tag clone was detected in bone, cartilage, or kidney in the mouse and human databases. Findings indicating the limited expression of Epfn in tooth germs are presented in Fig. S1. Furthermore, PTgs also showed strong immuno‐reactivity to the anti‐Epfn antibody, whereas thyroid glands did not (Fig. 1A–C). PCR analysis revealed the *Epfn* expression in PTgs and tooth buds, used as a positive control tissue for *Epfn* expression (Fig. 1D).

### Epfn KO mice show abnormal bone defects

**Fig. 2:**
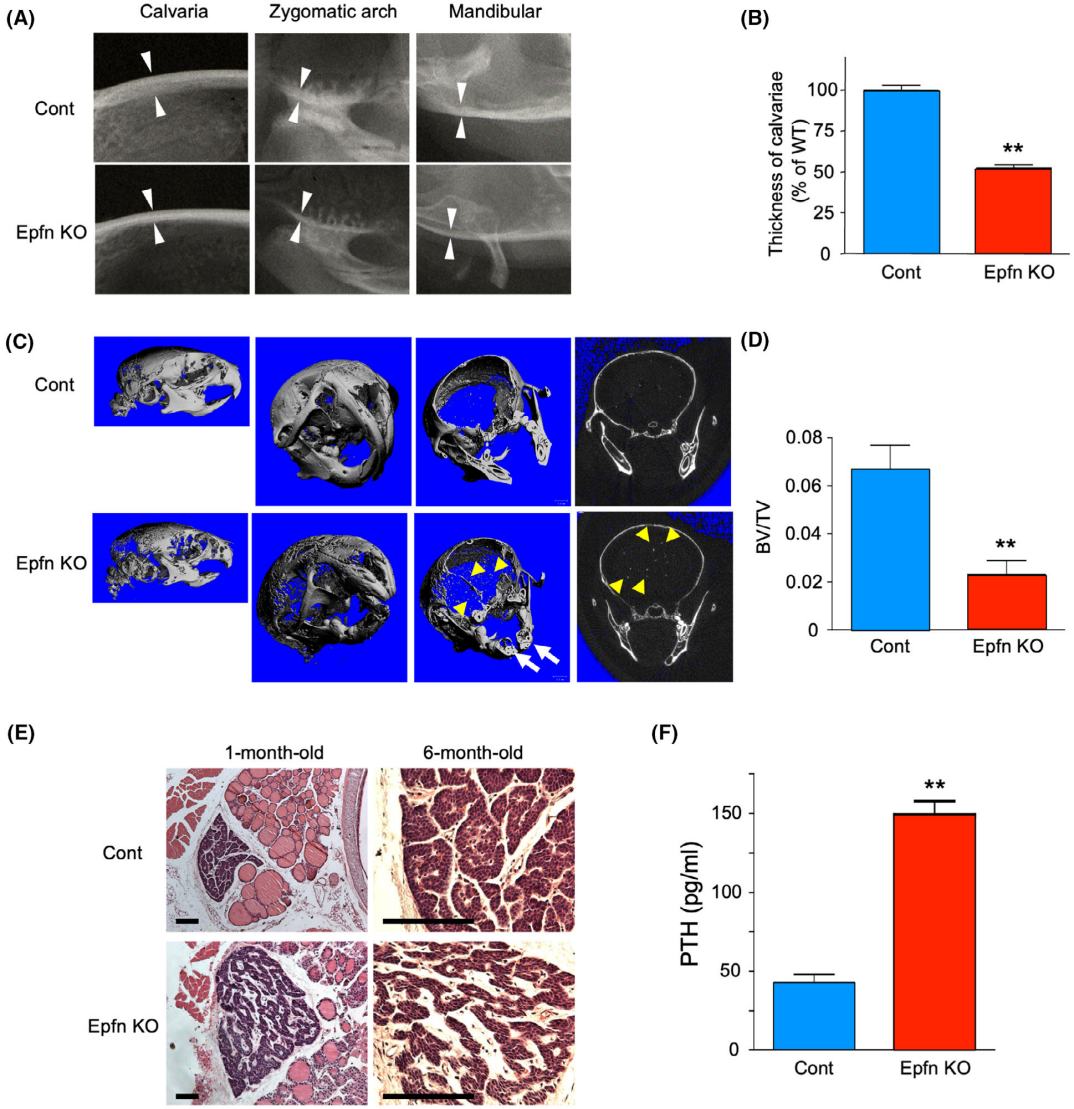
Abnormal skeletal and parathyroid phenotype in *Epfn* KO mice. (A) X‐ray images of the craniofacial bones of 3‐month‐old WT (Cont) and *Epfn* KO mice. Cortical bones in the calvaria, zygomatic arch, and mandibular inferior area were markedly thinner in *Epfn* KO mice. The apparent thickness of each skeletal element is indicated by opposing arrowheads. (B) WT (Cont) and *Epfn* KO calvaria thickness was determined at seven randomly selected points. Values are shown as the average value ± SD of three samples. (C) Three‐dimensional microCT images of WT (Cont) and *Epfn* KO mouse skulls. Right panels show side views and lower panels are transverse skull sections. Arrowheads indicate focal calcification of blood vessels and arrows indicate supernumerary teeth in KO mice. (D) Histomorphometric analysis of bone volume/total tissue volume (BV/TV) ratios of WT (Cont) and *Epfn* KO skulls. Values are shown as the average ± SD of three samples. (E) PTg tissues of 1‐month‐old and 6‐month‐old heterozygous (control, upper panels) and *Epfn* KO mice (lower panels). Bar indicates 100 μm (F) Serum PTH levels of 2‐week‐old *Epfn* heterozygous (cont) and *Epfn* KO mice. Values are shown as the average ± SD of four samples. **P < 0.01, as determined using the non‐parametric Mann–Whitney analysis (B, D, F).

A unique phenotype of *Epfn* knockout (KO) mice is supernumerary tooth formation due to dysregulation of proliferation and differentiation of dental epithelial cells [12]. When dissecting tooth buds from mandibular bones, a bone abnormality was noted in *Epfn* KO mice; thus, bone formation was analyzed. Radiography revealed that the craniofacial calvaria, zygomatic arch, and mandibular inferior cortical bones of the *Epfn* KO mice were much thinner than those of the wild‐type (WT) control mice (Fig. 2A,B; Cont vs. *Epfn* KO). Three‐dimensional image reconstruction and quantitative analysis using microCT confirmed cortical bone thinning in the KO mice (Fig. 2C,D). In addition, calcified particles were found scattered in the brain region (Fig. 2C, arrowheads), suggesting abnormal calcification of small vessels. *Epfn* expression was not noted in bone or the bone marrow gene expression libraries (Table S1), indicating that the bone phenotype may be a secondary effect of hyperparathyroidism due to lack of Epfn, which led to speculation that Epfn plays a role in the regulation of mineral metabolism. To identify the *in vivo* function of Epfn, we examined the histomorphology of PTgs in the *Epfn* KO mice. PTgs in 1‐month‐old *Epfn* KO mice were enlarged as compared to their heterozygous littermates (Fig. 2E; Cont vs. *Epfn* KO). Parathyroid epithelial cells were flattened in *Epfn* KO mice, while those in the controls were polygonal and showed a typical glandular structure (Fig. 2E). Disorganization of follicular cells resulted in altered squamous cell shapes, with the area of stromal tissue increased in enlarged *Epfn* KO parathyroid glands (Fig. 2E). Furthermore, the serum PTH level in the *Epfn* KO mice was approximately threefold greater than that in the control mice (Fig. 2F).

### Epfn negatively regulates PTH expression

**Fig. 3:**
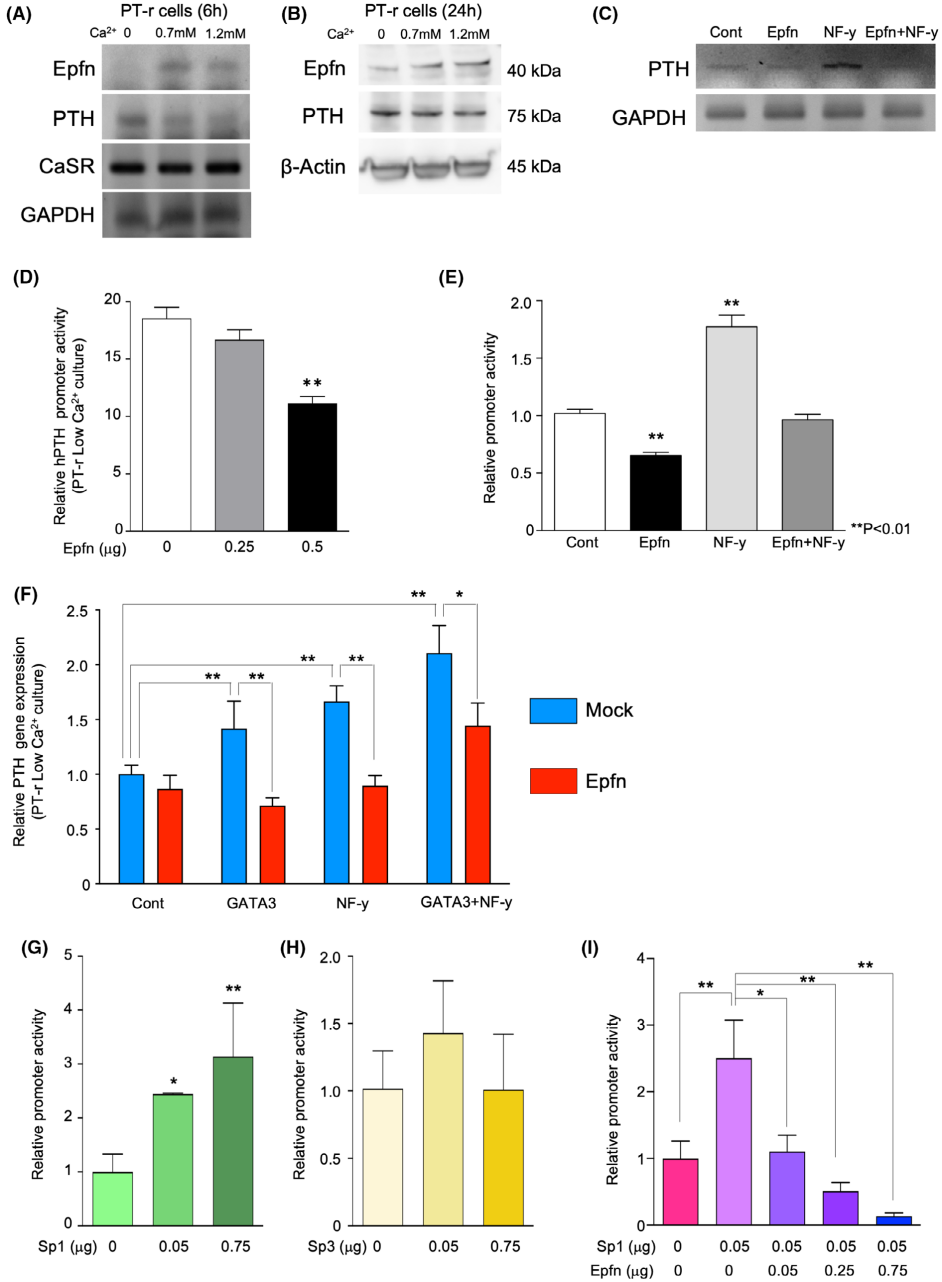
Regulation of *PTH* gene expression and promoter activity by Epfn. (A, B) PT‐r cells were maintained in low Ca^2+^ medium, or in the presence of 0.7 or 1.2 mm CaCl_2_ containing medium for 6 h (A) or 24 h (B). Total RNA and protein were purified at the end of the culture period and subjected to RT‐PCR and western blotting analysis respectively. Protein sizes are presented to the right of each band. Primer sequences are shown in Table S3. RT‐PCR and western blotting analysis were performed with three independent replicates. (C) RT‐PCR analysis of *PTH* gene expression in PT‐r cells transfected with the control, Epfn, NF‐y, or both expression vectors (Epfn plus NF‐y). RT‐PCR analysis was performed using three independent replicates. (D) Effect of Epfn on the human *PTH* promoter reporter. *PTH* promoter activity in PT‐r cells cultured in low Ca^2+^ medium was reduced in a dose‐dependent manner by increasing Epfn expression. Values are shown as the average ± SD of 6 wells. (E) Effect of Epfn and NF‐y expression on human *PTH* promoter reporter activity. *PTH* promoter activities in PT‐r cells cultured in low Ca^2+^ medium in the presence of 1.2 mm CaCl_2_ co‐transfected with the human *PTH* promoter reporter and the empty vector or Epfn, NF‐y, or Epfn plus NF‐y expression vectors. The luciferase activity was measured 24 h after transfection. Values are shown as the averages ± SD of 6 wells. (F) Real‐time PCR analysis of *PTH* gene in PT‐r cells transfected with either GATA3, NF‐y, or GATA3 + NF‐y with or without Epfn expression vector. PT‐r cell mRNA was extracted at 36 h after transfection. Values are shown as the averages ± SD of 3 wells. (G, H) The effect of Sp1 (G) or Sp3 (H) on the human *PTH* promoter reporter. *PTH* promoter activity in PT‐r cells cultured in low Ca^2+^ media was promoted in a dose‐dependent manner by the level of Sp1 expression, while no significant alteration was observed in Sp3 expression. Reporter assay was performed with 5 independent replicates. Values are averages ± SD of 6 wells. (I) The inhibition of Sp1 mediated *PTH* promoter activity by Epfn. Values are averages ± SD of 6 wells. **P < 0.01, *P < 0.05, as determined using the two‐way ANOVA multiple comparisons test (D–I).

To determine how Epfn regulates *PTH* gene expression, examinations were performed using cultures of a rat cell line (PT‐r) derived from PTgs [27]. A previous study found that PT‐r cells retain the calcium‐sensing mechanism following *PTH* transcription [28]. In the present experiments, PT‐r cells showed *PTH* expression in calcium‐depleted medium (Fig. 3A) and that was downregulated in a dose‐dependent manner when calcium ions were added (Fig. 3A, PTH), while *Epfn* expression was up‐regulated (Fig. 3A, Epfn). In contrast, the expression of *CaSR* was not affected by the concentration of calcium ions in the culture medium (Fig. 3A, CaSR). Consistent with gene expression analysis, Epfn protein expression was induced by increasing the concentration of calcium ions in the culture medium, while PTH expression was slightly reduced (Fig. 3B).

A conserved putative Sp binding site is located in the proximal promoter region of the *PTH* gene within 150 base pairs (bp) upstream of the transcriptional start site (Fig. S2A). This Sp binding site is adjacent to two nuclear factor‐y (NF‐y) sites that have key roles in *PTH* gene expression, and NF‐y and Sp1/3 have been shown to synergistically activate the *PTH* promoter [23, 29]. Sp1/3 also interact with GATA3, which binds in the proximal promoter region of the *PTH* gene, to promote *PTH* transcription [16]. NF‐y was found to stimulate *PTH* gene expression in PT‐r cells (Fig. 3C), as previously reported [23, 29], whereas Epfn did not alter the expression of *PTH*. On the other hand, co‐expression of Epfn and NF‐y in PT‐r cells, however, blocked stimulation of PTH gene expression, suggesting suppression of NF‐y‐induced *PTH* gene up‐regulation by Epfn in PT‐r cells (Fig. 2C, Epfn+NF‐y).

The human *PTH* promoter region was subdivided into the PGL4.72‐hRlucCP reporter plasmid, then a *PTH* promoter reporter construct was prepared (Fig. S2B) and reporter assays were carried out on PT‐r cultures. Reporter activity from the 1.3 kb human *PTH* promoter was stimulated in PT‐r cells cultured in the low Ca^2+^ medium (Fig. 3D) and was decreased by cotransfection with the Epfn expression vector (Fig. 3D). Transfection with NF‐y also increased the reporter activity (Fig. 2E, NF‐y), and this effect was counteracted by cotransfection with Epfn (Fig. 3E, Epfn+NF‐y). These findings indicate that Epfn is a suppressor of *PTH* gene transcription. Furthermore, we found that overexpression of either GATA3 or NF‐y alone, or both GATA3 and NF‐y, promoted the *PTH* gene expression in PT‐r cells cultured in the low Ca^2+^ medium (Fig. 3F). Similar to the promoter analysis findings, Epfn canceled GATA3‐, NF‐y‐, and GATA3 plus NF‐y‐mediated *PTH* expression in PT‐r cells (Fig. 3F). It has been shown that Sp1 and Sp3 also regulate the transcription of the *PTH* gene in parathyroid glands and cells [15, 16, 30]. In this study, overexpression of Sp1 was found to promote *PTH* promoter activity in PT‐r cells as previously demonstrated with other cell types (Fig. 3G) [30]. On the other hand, Sp3 did not show any *PTH* promoter activity in PT‐r cells (Fig. 3H). Next, we tested the suppression activity by Epfn against *PTH* gene induced by Sp1. Epfn dramatically canceled the Sp1‐mediated *PTH* promoter activity in PT‐r cells (Fig. 3I).

### Epfn interacts with the Sp binding site of the human PTH promoter

**Fig. 4:**
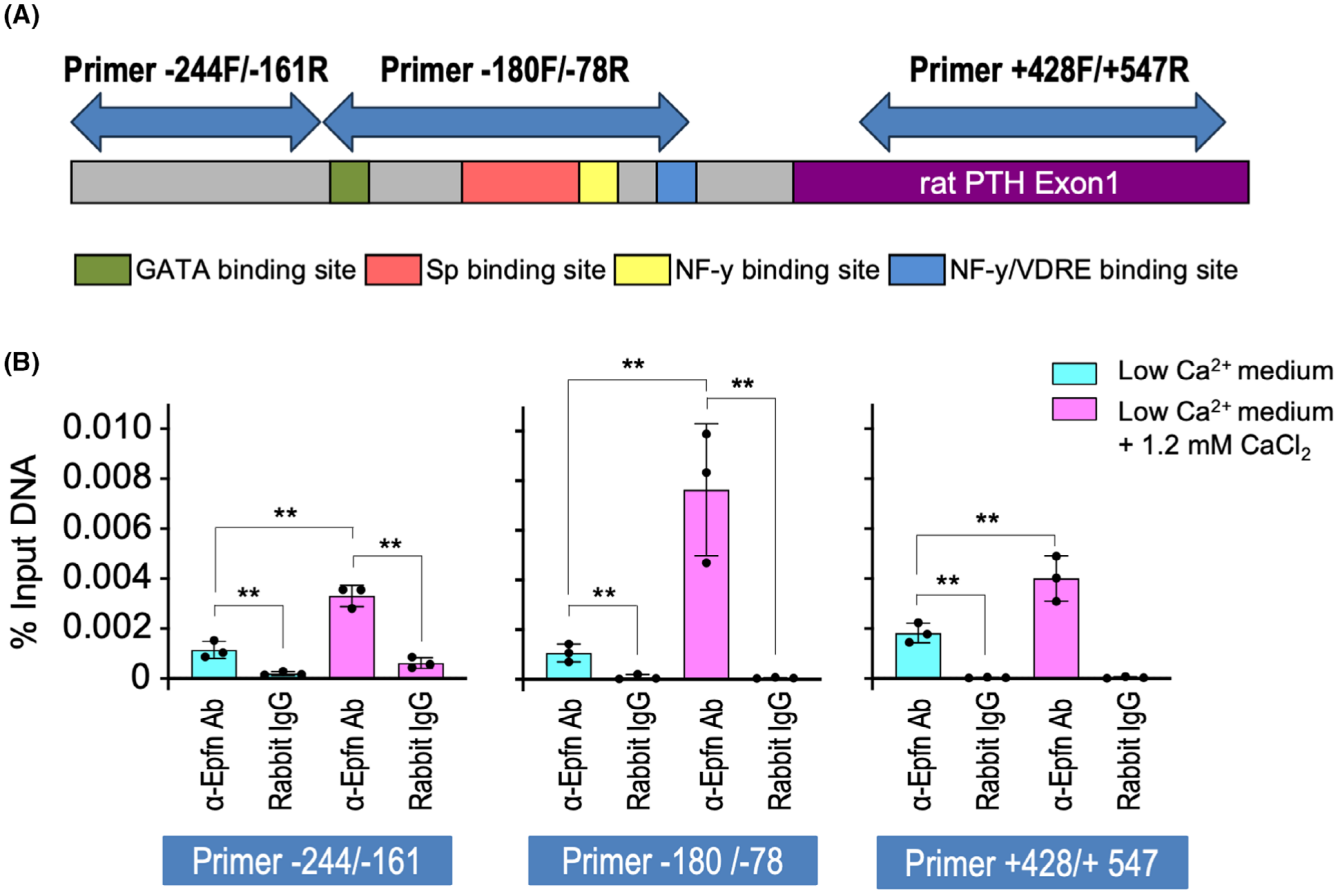
ChIP‐qPCR analysis of *PTH* promoter using anti‐Epfn antibody. (A) Schematic diagram of regions of PCR products shown by use of ChIP‐qPCR primer sets and GATA, Sp, NF‐y, and VDRE regulatory sites in the human *PTH* gene. (B) ChIP‐qPCR analysis of PT‐r cells maintained for 24 h in low Ca^2+^ medium (blue bar) or plus 1.2 mm CaCl_2_ containing medium (pink bar) using Epfn‐specific antibody (α‐Epfn Ab) or normal rabbit IgG (Rabbit IgG). Increased enrichment of immunoprecipitated DNA by the Epfn antibody was observed in PT‐r cells maintained in 1.2 mm CaCl_2_ containing medium with ‐180F/‐76 primer sets. ChIP‐qPCR analysis was performed with three independent experimental replicates. Values are shown as the averages ± SD of 3 wells. **P < 0.01, as determined using the two‐way ANOVA multiple comparisons test.

To identify conditions and regions of Epfn binding to the *PTH* promoter, chromatin immunoprecipitation (ChIP)‐qPCR analysis of PT‐r cells in the presence or absence of 1.2 mm CaCl_2_ was performed using Epfn‐specific antibody and normal rabbit IgG as a negative control for immunoprecipitation. Following sonication, the size of the average of chromatin in lysates from formaldehyde‐fixed PT‐r cells (1.3 × 10^7^ cells for each sample) was loaded onto a 1.5% agarose gel. An average size of 400 bp of chromatin lysate was used as input DNA and ChIP sample for each sample. Three primer sets were used to identify the three regions in the rat *PTH* promoter, including the Sp binding site (Fig. 4A). The significant binding of Epfn was detected under the low Ca^2+^ condition as compared with the samples immunoprecipitated with normal rabbit IgG (Fig. 4B, blue bar, α‐Epfn vs. Rabbit IgG). The binding of Epfn in the *PTH* promoter was significantly higher for all primer sets in PT‐r cells cultured under the high calcium condition compared to the cultures under the low calcium condition (Fig. 4B, pink bars vs. blue bars). The enrichment of Epfn was highly detected by the primer sets of −180/−78, which enriched the Sp binding site of the *PTH* proximal promoter as compared with the other primer sets (Fig. 4B). These results suggest that Epfn binds with the Sp binding region at the proximal human *PTH* promoter, and its binding activity of Epfn was increased in a high calcium culture condition in PT‐r cells.

### Epfn regulates PTH production

**Fig. 5:**
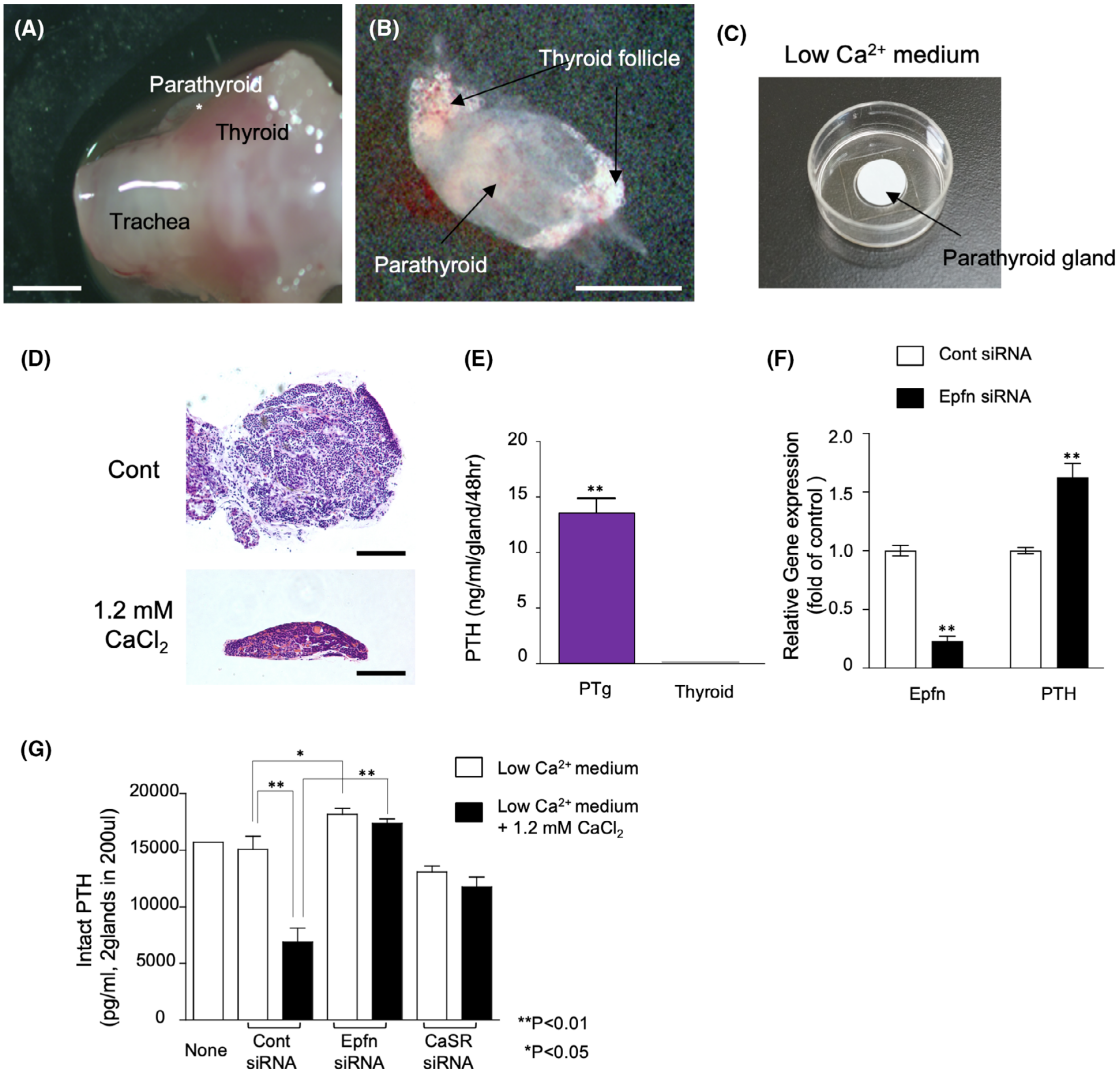
Mouse PTg *ex vivo* culture system and role of Epfn in PTH transcription and protein production. (A, B) Stereomicroscopic images of mouse trachea, thyroid, and PTg. Bar indicates 1 mm (A) and 250 μm (B) respectively. (C) *Ex vivo* mouse PTg culture system. Dissected mouse PTgs were cultured on a nucleopore filter membrane floating on low Ca^2+^ medium with 8% Ca^2+^‐reduced FBS. (D) HE‐stained sections of *ex vivo* parathyroid glands cultured for 48 h in low Ca^2+^ medium with 8% Ca^2+^‐reduced FBS in the presence or absence of 1.2 mm CaCl_2_. (E) Amount of PTH secreted by *ex vivo* PTgs cultured for 48 h in low Ca^2+^ medium with 8% Ca^2+^‐reduced FBS. Bar indicates 100 μm (F) Expressions of *Epfn* and *PTH* genes in control or *Epfn* siRNA‐treated PTgs cultured *ex vivo* for 24 h. (G) Effects of Epfn or CaSR silencing on PTH production in the PTg culture. PTg explants were transfected with either the control, *Epfn*, or *CaSR* siRNA for 24 h. Untreated control or siRNA‐treated PTg were cultured either in low Ca^2+^ medium with 8% Ca^2+^‐reduced FBS in the presence or absence of 1.2 mm CaCl_2_ for 24 h. The medium was then collected and subjected to an ELISA‐based PTH quantification assay. Values are shown as the averages ± SD of 6 samples. **P < 0.01, *P < 0.05, as determined using the non‐parametric Mann–Whitney analysis (E, F) or two‐way ANOVA multiple comparisons test (G).

To further examine the role of Epfn in PTgs, a murine PTg organ culture system was established. Normal PTgs were dissected together with thyroid glands and trachea (Fig. 5A), carefully isolated from other tissues with the use of a stereomicroscope (Fig. 5B). The samples were then cultured on filter paper in the inner well of a glass‐bottomed culture dishes (Fig. 5C) in low Ca^2+^ medium prepared from Ca^2+^‐free DMEM containing 8% Ca^2+^‐chelated fetal bovine serum (FBS) supplemented with 1 mm CaCl_2_. Under this condition, PTgs maintained the glandular structure (Fig. 5D) and continuously secreted PTH into the medium (Fig. 5E). When PTgs were cultured with a higher concentration of Ca^2+^ in the presence of 1.2 mm CaCl_2_ for 2 days, they shrank and PTH production was reduced (data not shown). In PTg cultures treated with *Epfn* siRNA, Epfn expression was reduced to 30% of the controls treated with scrambled siRNA (Fig. 5F, Epfn), while *PTH* gene expression was increased by approximately 1.5‐fold (Fig. 5F, PTH). When the control PTg cultures were cultured with a higher concentration medium in the presence of 1.2 mm CaCl_2_, the production of PTH was reduced by half (Fig. 5G; Cont siRNA), but this downregulation was diminished by silencing Epfn or CaSR (Fig. 5G). Together, these results indicate that Epfn expression is required for the inhibition of PTH production by PTgs in response to increased extracellular calcium levels and that Epfn may function as a downstream molecule of CaSR signaling.

### Reduced expression of Epfn in parathyroid adenomas

**Fig. 6:**
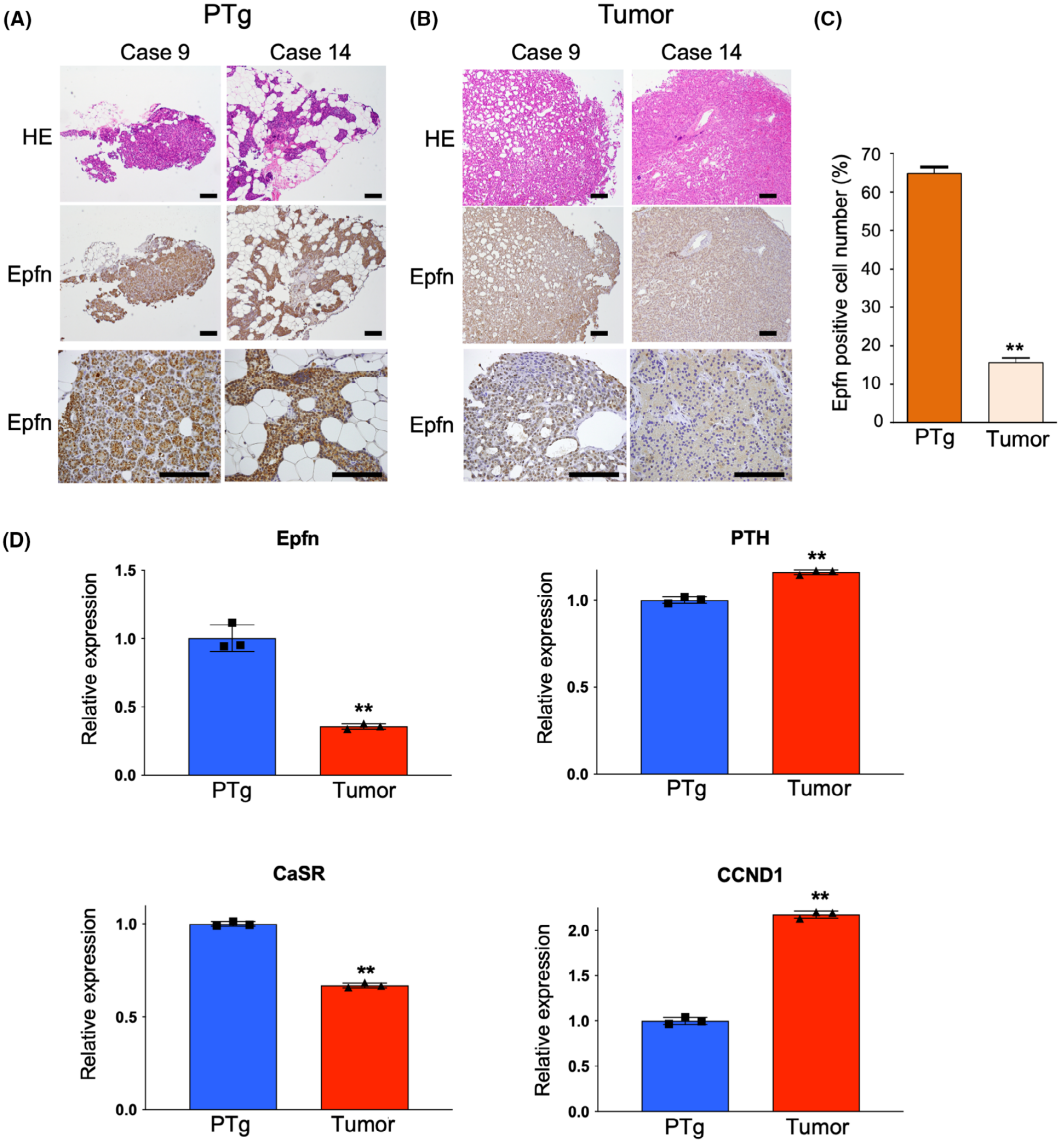
Reduced expression of Epfn in the parathyroid glands of PHPT patients. PTgs and parathyroid tumors were obtained from PHPT patients (case 9 and 14). (A–C) Sections of PTgs (A) and parathyroid tumors (B) were stained with HE or Epfn antibody (Epfn). Bar indicates 100 μm (C) Percentage of Epfn‐positive cells in normal parathyroid and parathyroid hyperplasia. (D) RT‐qPCR analysis of *Epfn*, *PTH*, *CaSR*, and *CCND1* in parathyroid glands (PTg) and adenoma (Tumor) from PHPT patient (case 14). Immunohistochemistry and RT‐qPCR analysis were performed with three independent replicates. Values are shown as the averages ± SD. **P < 0.01, as determined using the non‐parametric Mann–Whitney analysis (C, D).

To extend the results obtained in animal models to better understand related diseases in humans, parathyroid tissues were obtained from 12 patients diagnosed with hyperparathyroidism (PHPT) (Table S2). The clinical research protocol was approved by the Ethics Committee of Tohoku University. Additionally, samples of normal parathyroid tissues near tumors were also obtained from the patients. A total of 11 specimens were identified as parathyroid adenoma and composed of parathyroid cells lacking stromal adipose cells (Fig. 6B, HE), while normal PTgs contained both types of cells and displayed a glandular structure (Fig. 6A). Immunostaining with the anti‐Epfn antibody revealed that more than half of the chief cells in the PTgs were positive (Fig. 6A, Epfn), whereas the parathyroid adenoma specimens in all cases had much lower immunoreactivity against Epfn (Fig. 6A–C). To compare gene expressions between the parathyroid adenomas and parathyroid glands, we performed RT‐qPCR analysis in case 14 patient. In concordance with immunohistochemistry results, *Epfn* gene expression was increased in the parathyroid gland and inversely decreased in the adenoma, while the expressions of *PTH* and *CCND1* were upregulated, and interestingly, the expression of *CaSR* was diminished in the adenoma (Fig. 6D). These findings were consistent with the present experimental results and were considered to indicate that the negative regulation of *PTH* expression in a parathyroid adenoma is related to decreased expression of *CaSR* and *Epfn*.

### Regulation of Epfn expression by Nfatc2 and calcimimetic agent R‐568

**Fig. 7:**
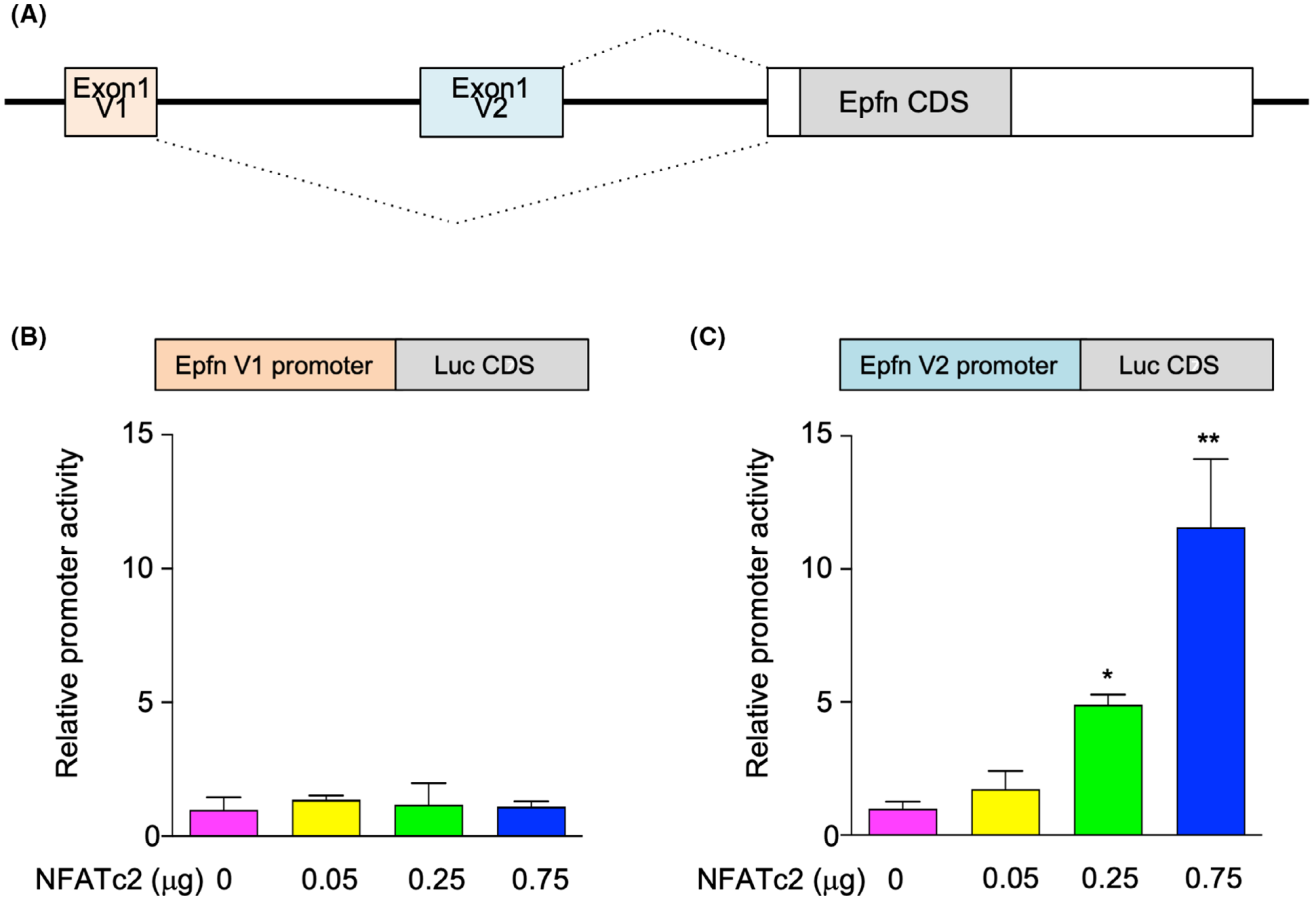
Gene structure of *Epfn* and Epfn induction by Nfatc2. (A) Two isoforms of *Epfn* transcription gene. (B, C) Luciferase promoter reporter constructs containing variant 1 exon1 promoter (B) and variant 2 promoter (C). Promoter activity for the variant 2 isoform was stimulated in a dose‐dependent manner by increasing the amount of the Nfatc2‐expressing vector. Values are shown as the averages ± SD of 6 wells. **P < 0.01, *P < 0.05, as determined using the two‐way ANOVA multiple comparisons test (B, C).

The present findings indicate that Epfn is a novel transcription factor that regulates *PTH* gene expression in response to serum Ca^2+^ levels. Finally, regulation of Enfn transcription by an increase in extracellular serum Ca^2+^ level followed by upregulation of cytoplasmic Ca^2+^ was examined. The NFAT family (NFAT1‐4) is activated by cytoplasmic Ca^2+^ [31, 32]. The *Epfn* transcript is regulated by the two distinct putative promoters (Fig. 7A) [1]; thus, *Epfn* promoter luciferase vectors, shown to contain 1000‐bp putative *Epfn* promoters (Epfn V1 and V2), were constructed (Fig. 7B,C). Experiments were then performed to examine Nfatc2 (NFAT1) as a major regulator of NFAT signaling in PTgs [33, 34]. Nfatc2 did not activate the Epfn V1 promoter (Fig. 7B), whereas the Epfn V2 promoter was activated in an Nfatc2 vector concentration‐dependent manner. In support of those findings, an Nfat binding sequence was noted in the Epfn V2 promoter region, while no such binding sequence was found in the proximal Epfn V1 promoter (Fig. S3A,B), indicating that induction of Epfn in response to serum Ca^2+^ levels is controlled by Nfatc2.

In European countries, cinacalcet is used for treatment of intractable hypercalcemia in patients with PHPT [35]. Cinacalcet is a calcimimetic agent that increases the calcium sensitivity of CaSRs in PTgs, resulting in a reduction of serum PTH concentration [36, 37]. Experiments were performed to determine whether Epfn is involved in the mechanism by which calcimimetic agents suppress *PTH* transcription. The results showed that R‐568, another calcimimetic agent, promoted Epfn gene expression (Fig. S4) and decreased PTH production in PT‐r cells within 12 h (data not shown), suggesting that Epfn is a mediator of the pharmacological action of this calcimimetic agent.

**Fig. 8:**
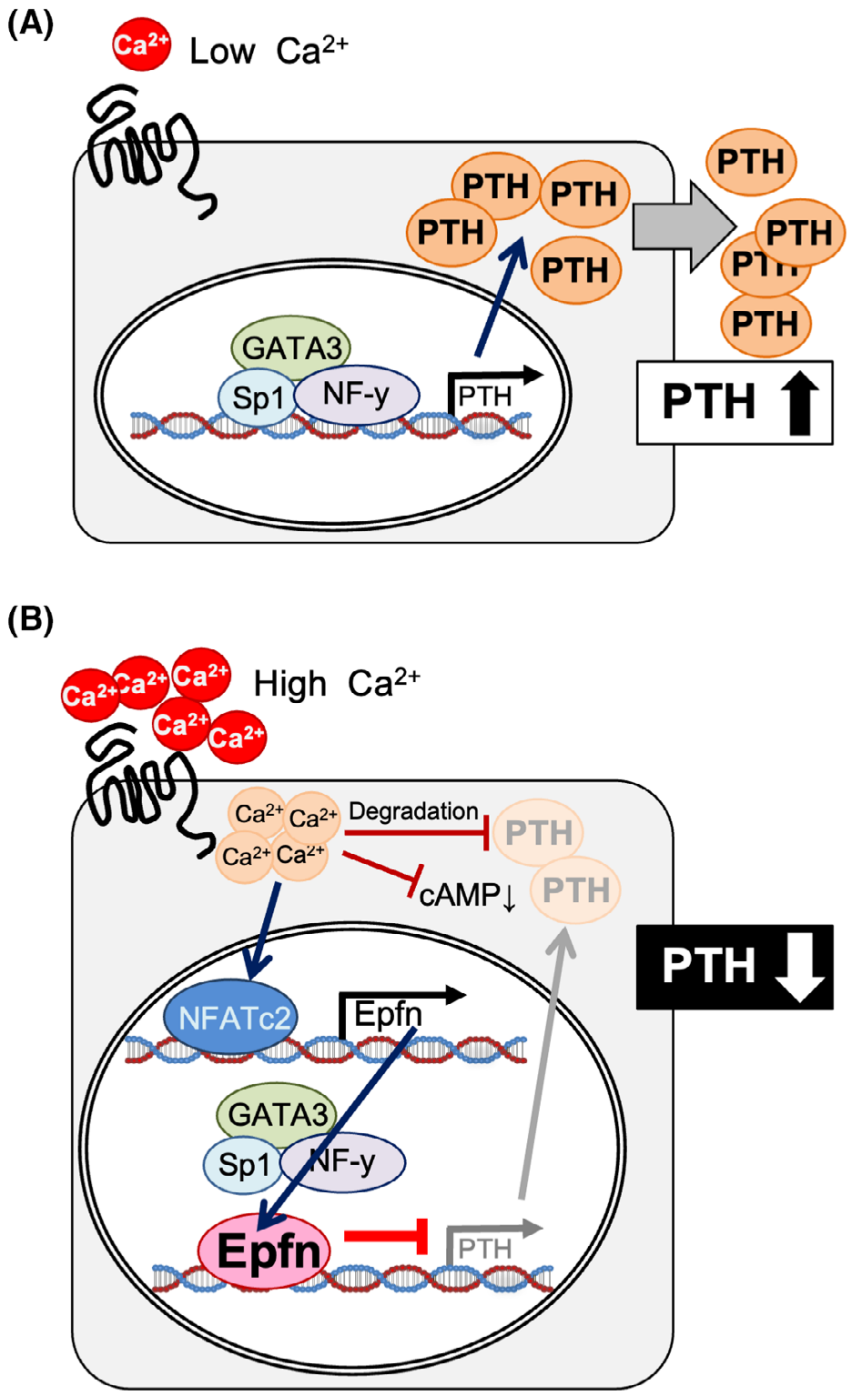
Proposed model of Epfn function in parathyroid glands. Upper panel. With a low concentration of serum Ca^2+^, *PTH* gene transcription is promoted by GATA3, Sp1, and NF‐y. Lower panel. With a high concentration of serum Ca^2+^, CaSR is activated in parathyroid cells, followed by an increase of cytoplasmic Ca^2+^. Cytoplasmic Ca^2+^induces degradation of the *PTH* gene and downregulates cAMP, and also activates NFAT signaling. Nfatc2 stimulates the *Epfn* transcript by binding the variant 2 promoter NFAT responsive element. Induced Epfn blocks activation of *PTH* gene transcription by GATA3, Sp1, or NF‐y.

Shown in Fig. 8 is a proposed mechanism for control of *PTH* gene expression based on the level of serum Ca^2+^, which occurs in part through Epfn and its transcriptional regulation. The present findings related to the function of Epfn may contribute to the development of novel drugs for treating patients with primary as well as secondary hyperparathyroidism.

## Discussion

The present findings indicate that Epiprofin (Epfn), a transcription factor expressed in the teeth, hair, and epithelium, is also expressed in the parathyroid glands, induced in response to an increase in extracellular calcium concentration. It has been postulated that Epfn plays a novel role in the regulation of parathyroid hormone (PTH) secretion in calcium homeostasis. We also found that Epfn negatively regulates the transcription of the *PTH* gene, possibly through binding to the Sp site in the proximal promoter region of the *PTH* gene, indicating Epfn as a novel transcription factor that regulates *PTH* gene expression and calcium homeostasis.

Experiments with cultured cells and organ cultures of the parathyroid gland demonstrated that Epfn is upregulated in response to the extracellular calcium concentration and negatively regulates *PTH* gene expression. Consequently, observations of parathyroid tissues from Epfn knockout mice revealed that they were larger in size and secreted more PTH than normal mice. Elevated serum PTH from enlarged parathyroid glands has been reported in several mouse models, including deficiencies in *CaSR*, *Cyp27b1*, *MEM1*, and *VDR*, and parathyroid‐targeted overexpression of *cyclin D1* transgenic mice [35, 36, 37, 38, 39]. Including *Epfn* KO mice, each of these mouse models shows phenotypes that partially resemble human familial hyperparathyroidism. In the histological analysis of the parathyroid glands of these mutant mice, the *MEM1* cKO with *PTH‐Cre* and *PTH‐CyclinD1* transgenic mice showed changes in the cell shape of the follicular cells and enlarged stromal tissue similar to those observed in the *Epfn* KO mice [37, 39]. On the other hand, the follicular cell shapes have been reported to be normal in *CaSR*, *Cyp27b1*, and *VDR* KO mice [35, 36, 38]. The *Epfn* KO, *MEM1* cKO, and *PTH*‐*CyclinD1* TG mouse models are associated with hypercalcemia, while the *CaSR*, *Cyp27b1*, and *VDR* KO mouse models have a normal serum calcium level despite elevated PTH due to impaired calcium absorption in the kidneys and intestine. Taken together, it is speculated that hypercalcemia results in the characteristic parathyroid histology of *Epfn* KO mice.

Understanding of the present results of *PTH* promoter analysis showing a low calcium condition associated with Epfn in parathyroid gland cells is very important. Luciferase analysis of the *Epfn*‐forced‐expression system showed that Epfn suppressed *PTH* promoter activity in cells cultured in low calcium medium, and even more in the presence of GATA3 and NFy, which function to activate the *PTH* promoter. These results indicate that Epfn might be an essential negative regulator for *PTH* transcription, even in a low calcium condition. Epfn may therefore be a drug target factor potentially capable of inhibiting *PTH* transcription even in hypocalcemic conditions, such as in patients with secondary hypoparathyroidism.

The present experiments also demonstrated that the forced expression of Epfn blocks the *PTH* transcription, even under a low Ca^2+^ condition in PT‐r cells as well as in a PTg organ culture system, and Epfn interacts with a highly conserved Sp1 DNA element in the *PTH* promoter region. Sp1 and Sp3 have been reported as candidate regulators of *PTH* transcription [15]. Since PTH is expressed only in the parathyroid glands, it is reasonable to assume that the transcriptional regulation of PTH involves tissue‐specific factors, such as Epfn, rather than ubiquitous transcription factors, such as Sp1 or Sp3. It is thus considered that the development of novel therapies targeting Epfn, which is only expressed in tissues, may also contribute to minimizing side‐effects. Based on the results obtained, it is considered that the forced expression of Epfn could be used to treat secondary hyperparathyroidism in patients with chronic kidney disease (CKD).

Cinacalcet is a calcimimetic drug approved in the United States for treatment of secondary hyperparathyroidism in patients with CKD receiving dialysis and also hypercalcemia in patients with parathyroid carcinoma. It is widely used worldwide by clinical nephrologists for treatment of secondary hyperparathyroidism and hypercalcemia due to parathyroid carcinoma. However, cinacalcet is not always effective in all hyperparathyroidism patients, such as those with congenital *CaSR* mutations causing familial hypocalciuric hypercalcemia or neonatal hypoparathyroidism [40, 41]. Indeed, findings of the present gene expression analyses of PHPT patients showed the downregulation of the *CaSR* gene (Fig. 6D), indicating a reduced response to extracellular Ca^2+^ level as well as to cinacalcet. In such patients, the reduced sensitivity to serum calcium due to reduced *CaSR* expression and/or function means that cinacalcet is not expected to have a lowering effect on serum PTH levels. Furthermore, cinacalcet binds to CaSR not only in the PTgs but also in other organs, including those in the cardiovascular system, possibly causing side effects in the circulatory system [42]. Our findings showing that Epfn is an effector of CaSR signaling, thus reducing PTH production, suggest that control of Epfn expression could be a novel therapeutic strategy for such cases.

Transcriptional regulation of Epfn in PTg tumor cells could be one of the most important issues for developing a translational strategy to treat PHPT. As the present findings showed, Epfn is induced by increased extracellular Ca^2+^ concentration in parathyroid cells. This phenomenon, the extracellular Ca^2+^ mediated induction of *Epfn* expression, is also observed in other cell types such as epidermal keratinocytes, ameloblasts, and odontoblasts. The present findings also demonstrated that the induction of *Epfn* transcription by an increase in extracellular Ca^2+^ regulates the activation of Nfatc2. Interestingly, the activation of dephosphorylated‐Nfatc1 was previously found to promote the expression of Epfn in keratinocytes [43]. Nfatc1 is a member of the NFAT family and shows a high degree of (> 80%) sequence similarity in the DNA‐binding motif with Nfatc2. Thus, it is possible that Nfatc2 shares the DNA‐binding domain and regulatory regions with Nfatc1 in the *Epfn* promoter [44]. Additionally, we previously reported the Epfn binding sites in the Nfatc2 promoter region by ChIP‐seq analysis (GSE145909) [4], suggesting that Epfn also upregulates *Nfatc2* expression. It is possible that the *Epfn* and *NFAT* gene expression regulatory systems may interact with each other. It has been suggested that the regulation of *Epfn* expression by NFAT is a common mechanism in several cells; we will continue to analyze NFAT binding sites in the *Epfn* promoter region.

In addition to CaSR and VDR activation, the FGF23‐Klotho endocrine axis is important for the negative regulation of PTH production [45]. In parathyroid cells, FGF23 binds to the FGFR‐Klotho complex to suppress PTH production by activating the MAPK‐pathway [45]. Recently, Nfatc2 has been identified as a critical regulator in Klotho‐independent PTH suppression [34]. In this suppression axis, Epfn is likely involved in the suppression of PTH production.

In summary, the present results provide several lines of evidence that Epfn is a negative regulator of PTH production, acting on events that occur downstream of extracellular Ca^2+^ sensing by CaSR. Therefore, Epfn is a potential therapeutic target for control of PTH production in primary and secondary hyperparathyroidism patients with CKD.

## Materials and methods

### Mice

*Epfn* KO mice in inbred C57BL/6 background were generated as previously described [12]. This study was performed in accordance with the National Institute of Health guidelines for the use of experimental animals, and all animal protocols have been approved by the Institutional Animal Care and Use Committee (IACUC) (IACUC: 2020DnA‐016‐07). The mice were housed in an animal facility approved by the American Association for the Accreditation of Laboratory Animal Care. For μCT examinations, PTH measurements, and histological analyses, we used at least 4 independent sets of Epfn KO male mice and control mice. Wild‐type (C57BL/6) or heterozygous male littermates were used as controls. Euthanasia was performed by cervical dislocation under general anesthesia.

### X‐ray and μ‐CT analyses

Tissue samples were fixed in 4% paraformaldehyde at 4 °C, then subjected to micro‐CT analysis using a CT40 scanner (SCANCO Medical, Brüttisellen, Switzerland) at 55 kV and 70 mA. Data were analyzed at a threshold of 244 for detection of mineralized components and calculation of bone volume (BV), and then re‐analyzed at a threshold of 110 to determine total sample volume (TV). Normalized bone volume/tissue volume ratio (BV/TV) was calculated by dividing BV by TV, and used to validate differences between experimental and control values. X‐ray imaging was performed with a piXarray100 (Bioptics, Tucson, AL, USA) in auto‐exposure mode. Three independent groups of *Epfn* KO and control mice were analyzed, with tissues from WT littermates used as controls.

### Cell culture and transfection

The PT‐r rat parathyroid cell line was kindly gifted from Dr. K. Sakaguchi (Wakayama Medical University) [28]. PT‐r cells were maintained in DMEM/F‐12 (Invitrogen, Carlsbad, CA, USA) supplemented with 10% FBS that was treated with chelex‐100 resin (BioRad, Hercules, CA, USA) to remove free Ca^2+^ and confirmed to be mycoplasma‐free throughout the culture process. A reverse transfection technique was utilized to transfect PT‐r cells with plasmid DNA using Lipofectamine LTX with PLUS Reagent (Thermo Fisher, Carlsbad, CA, USA). A mixture of Lipofectamine LTX reagent at 1 μL was mixed with 25 μL of Opti‐MEM medium (Invitrogen), and 1 μL of PLUS reagent was mixed with 25 μL of Opti‐MEM medium containing 250 or 500 ng of plasmid DNA, with each mixture separately incubated for 10 min at room temperature. The two solutions were mixed and incubated for 20 min at room temperature in 48‐well plates to which 3.0 × 10^4^ of PT‐r cells were then added per well. Cells were cultured for 48 h before processing for assays.

### Western blotting

PT‐r cells were grown in DMEM/F‐12 supplemented with 10% chelex‐100‐treated FBS in 100 mm dishes at a density of 3.0 × 10^4^ per cm^2^, with 0.7 or 1.2 mm of CaCl_2_, or the same volume of water added to the culture medium for 24 h. Cells were washed twice with ice‐cold PBS and lysed with CytoBuster Protein Extraction Reagent (Merck, Darmstadt, Germany) supplemented with a proteinase inhibitor cocktail (Sigma‐Aldrich, St. Louis, MO, USA). After centrifugation, the supernatants of cell lysis samples were transferred to fresh tubes and were separated using 4–12% SDS/PAGE (NuPAGE, Thermo Fisher) and analyzed by western blotting. The blotted membranes were incubated with antibodies for Epfn (1 : 1000, #21234‐1‐AP, ProteinTech, Rosemont, IL, USA), PTH (1 : 50, 7170‐6216; AbD Serotec, Marnes‐la‐Coquette, France), or beta‐actin (1 : 1000, #5125, Cell Signaling Technology, Danvers, MA, USA), then detected with an Immobilon ECL kit (Merck) and visualized using an ImageQuant LAS 4000 Mini image analysis system (GE Healthcare Life Science, Chicago, IL, USA).

### PTH reporter construction and assay

The human *PTH* promoter was amplified with KOD DNA polymerase (Toyobo, Osaka, Japan) using the primers 5′‐GGCGGTACCTCTAAAATGGAGCCTGGAGCAAC‐3′ and 5′‐CCGCTCGAGAACTAAAGACAACTGATGAATTGGACTGCA‐3′. A 1.3‐kb PTH fragment was then subcloned into the Acc651/Xho I site of pGL4.72‐hRlucCP (Promega, Madison, WI, USA). Renilla luciferase and firefly luciferase activities were determined using a Dual‐Luciferase Reporter Assay Kit (Promega), according to the manufacturer's protocol, and a Turner Design 20/20 luminometer (Promega). Reporter assays were performed using five independent replicates.

### Histology and immunohistochemistry

Parathyroid glands were dissected from 1‐ and 6‐month‐old mice, fixed with 4% paraformaldehyde in PBS overnight at 4 °C, and embedded in paraffin. Sections (6 μm) were prepared and stained with Harris hematoxylin and eosin Y. For Epfn immunostaining, the tissue sections were incubated for 30 min in 0.3% (v/v) hydrogen peroxide in methanol to block endogenous peroxidase activity. Sections were blocked in 1% normal donkey serum in 0.5% Triton‐X100 in PBS (TSP) for 1 h at 37 °C. After overnight incubation at 4 °C with the affinity‐purified rabbit polyclonal anti‐Epfn antibody (1 : 300) [1, 12] or anti‐Epfn antibody (1 : 300, #21234‐1‐AP, ProteinTech), the antibodies were detected with HRP‐conjugated anti‐rabbit IgG (N‐Histofine; Nichirei, Tokyo, Japan), followed by development with 3,3′‐diaminobenzidine (DAB) hydrochloride (Histofine Simple Stain DAB; Nichirei). Counterstaining was performed using Harris hematoxylin diluted 1 : 10. For immunofluorescent analysis, antibodies were detected with Alexa594‐conjugated secondary antibodies (ThermoFisher) and cell nuclei were stained with DAPI (Dojindo, Kumamoto, Japan). Immunofluorescent images were captured with a Nikon Ni‐E microscope with an Andor Zyla camera and analyzed using the Nikon NIS Elements software package. Three independent examinations of Epfn KO and control mice were conducted, with WT littermates used as the controls. For clinical samples, immunohistochemistry examinations using three serial sections per specimen were performed.

### RNA isolation, reverse transcription, PCR, and real‐time PCR

Total RNA was isolated using TRIzol (Invitrogen) and the samples were treated with RNase‐free DNase I (Nippon Gene, Tokyo, Japan). First‐strand cDNA was synthesized at 42 °C for 50 min using a SuperScript VILO cDNA Synthesis Kit (Invitrogen). PCR reactions were performed using the Takara Ex Taq HotStart Version (Takara, Otsu, Japan) or SYBR Green PCR Master Mix (Applied Biosystems, Carlsbad, CA, USA). Total RNA samples were also prepared from normal and tumorous PTg tissues obtained from the PHPT patients and subjected to quantitative PCR to determine *Epfn* and *CaSR* gene expression levels. Relative gene expression levels were calculated by subtracting the Ct value of the target gene from that of *GAPDH* for each patient. Primer sequences are listed in Table S3.

### ChIP assay

A ChIP‐IT High Sensitivity kit (#53040 Active Motif) was used to determine the associations of Epfn with the genomic regions on the proximal promoter of the *PTH* gene. Antibodies used for ChIP included anti‐Sp6 (#21234‐1‐AP, ProteinTech) and Normal Rabbit IgG (#2729; Cell Signaling Technology), both at 4 μg. PT‐r cells were cultured in the presence or absence of 1.2 mm of CaCl_2_ for 24 h. Chromatin in formaldehyde‐fixed PT‐r cell lysates (1.3 × 10^7^ cells) was sonicated to an average size of 400 bp by using a VP‐300N ultrasonicator (TAITEC, Saitama, Japan) for 20 min at 25% amplitude (30 s on/off) on ice. ChIP‐qPCR was performed using a BioRad CFX96 Touch Real‐Time PCR System, and the enrichment of gene promoter fragments was normalized to its respective input DNA value. Primer sequences used for qPCR are listed in Table S3.

### *Ex vivo* culture of PTgs

*Ex vivo* PTg culturing was performed using a previously described method, with some modification [46]. PTgs were dissected from 4‐week‐old ICR mice, and 10 PTgs/group were cultured on a Whatman Nucleopore Track‐Etched Filter (13 mm, 0.1 μm pore size, Whatman, Maidstone, UK) placed in an inner well of a 35‐mm glass‐bottomed microwell dish (MatTek, Ashland, MA, USA). FBS used for the cultures was treated with Chelex‐100 resin (BioRad, cat # 1422832) to reduce free Ca^2+^ [47]. PTgs were maintained in low Ca^2+^ medium prepared from Ca^2+^‐free DMEM containing 8% of Ca^2+^‐chelated FBS supplemented with 1 mm CaCl_2_ and 100 U·mL^−1^ penicillin and 100 μg·mL^−1^ streptomycin. For the high Ca^2+^ condition, 1.2 mm CaCl_2_ was added to PTg growth medium and cultured for 24–48 h.

### Measurement of PTH production

The level of PTH in 200 μL of PTg culture media or mouse sera was determined using a Mouse Intact PTH ELISA kit (Immutopics, Inc., San Clemente, CA, USA). Following euthanasia, blood samples were collected from the heart apex and stored on ice, then subjected to centrifugation at 1000 ***g*** for 10 min at 4 °C to collect serum samples. At least 4 independent specimens were investigated, with WT littermates used to supply controls.

### Small RNA interference

siRNAs for *Epfn* and *CaSR* were obtained from QIAGEN (mouse SP6; SI01430016 and SI01429995, mouse CaSR; SI02740283 and SI00208033, QIAGEN, Valencia, CA, USA), then PTg *ex vivo* cultures were transfected with 250 nm of siRNA using Oligofectamine reagent (Invitrogen). Total RNA was purified after 24 h of culture, and gene silencing was confirmed by real‐time PCR analysis.

### Clinical samples

The use of the human samples was approved by the Ethics Committee of Tohoku University (approval number: 2011‐159 and 23‐10), and experiments were undertaken with the understanding and written consent of each subject from all participants (*n* = 23). The study methodologies conformed to the standards set by the Declaration of Helsinki. The timeframe during which samples were collected (September 2011 to October 2019). All of the patients received a clinical diagnosis of PHPT and had not undergone prior related therapy. The 2014 guidelines were followed for diagnosis and PHPT treatment strategy in all cases [48]. Specimens were collected during surgery at Tohoku University Hospital. Donor anonymity was maintained.

### Statistical analysis

Differences between variables were assessed using non‐parametric Mann–Whitney analysis or two‐way ANOVA multiple comparisons test (GraphPad Prism 8 software, GraphPad Prism, Boston MA, USA; RRID:SCR_002798). The Spearman correlation coefficient was used to determine the expression level of Epfn (Epfn/GAPDH) as compared with that of serum Ca^2+^, total PTH level, and PTg tumor weight. Each calculation was performed three times to test the reproducibility of the results for the cell culture and organ culture experiments, with representative findings presented.

## Conflicts of interest

The authors declare no conflict of interest.

## Author contributions

TN and MI designed the project and wrote the manuscript. ME‐I and MP helped with drafting the manuscript. For the experiments, NN, HMN, SF, and MI performed gene and protein expression analysis and quantification; TN and MI established the PTg explant cultures; YI and TN performed promoter analysis; NN, MW, and TN analyzed the human specimens.

## Peer review

The peer review history for this article is available at https://www.webofscience.com/api/gateway/wos/peer‐review/10.1111/febs.70085.

## Supporting information


**Fig. S1.** Immunohistochemical analysis of Epfn using anti‐Epfn antibody in mouse tooth germs.
**Fig. S2.** Sequence similarity and conservation of transcription factor responsive elements in PTH proximal promoter region among species.
**Fig. S3.** DNA sequences of Epfn promoter variants.
**Fig. S4.** RT‐qPCR analysis of Epfn expression in PT‐r cells cultured with R‐568.
**Table S1.** Expression profile of Epfn expression in sequence tag clone in NCBI UniGene.
**Table S2.** Summary of PHPT patient age, blood test results, and histological diagnosis.
**Table S3.** Primers used in this study.

## Data Availability

All data generated or analyzed during the present study are included in the published article. The ChIP‐seq dataset of Epfn peaks from both mouse molar epithelial and mesenchyme samples has been deposited as GEO accession GSE145909.
