# Supplementary material for: Molecular mechanism for transcriptional regulation of the parathyroid hormone gene by Epiprofin
Source: FEBS J. 2025 Mar 31;292(14):3659–75. doi: 10.1111/febs.70085 (PMC12265863; doi:10.1111/febs.70085)
Supplement: Supplementary file 1 — Fig. S1. Immunohistochemical analysis of Epfn using anti‐Epfn antibody in mouse tooth germs. Fig. S2. Sequence similarity and conservation of transcription factor responsive elements in PTH proximal promoter region among species. Fig. S3. DNA sequences of Epfn promoter variants. Fig. S4. RT‐qPCR analysis of Epfn expression in PT‐r cells cultured with R‐568. Table S1. Expression profile of Epfn expression in sequence tag clone in NCBI UniGene. Table S2. Summary of PHPT patient age, blood test results, and histological diagnosis. Table S3. Primers used in this study. [file FEBS-292-3659-s001.pdf]

Supplemental Figure 1

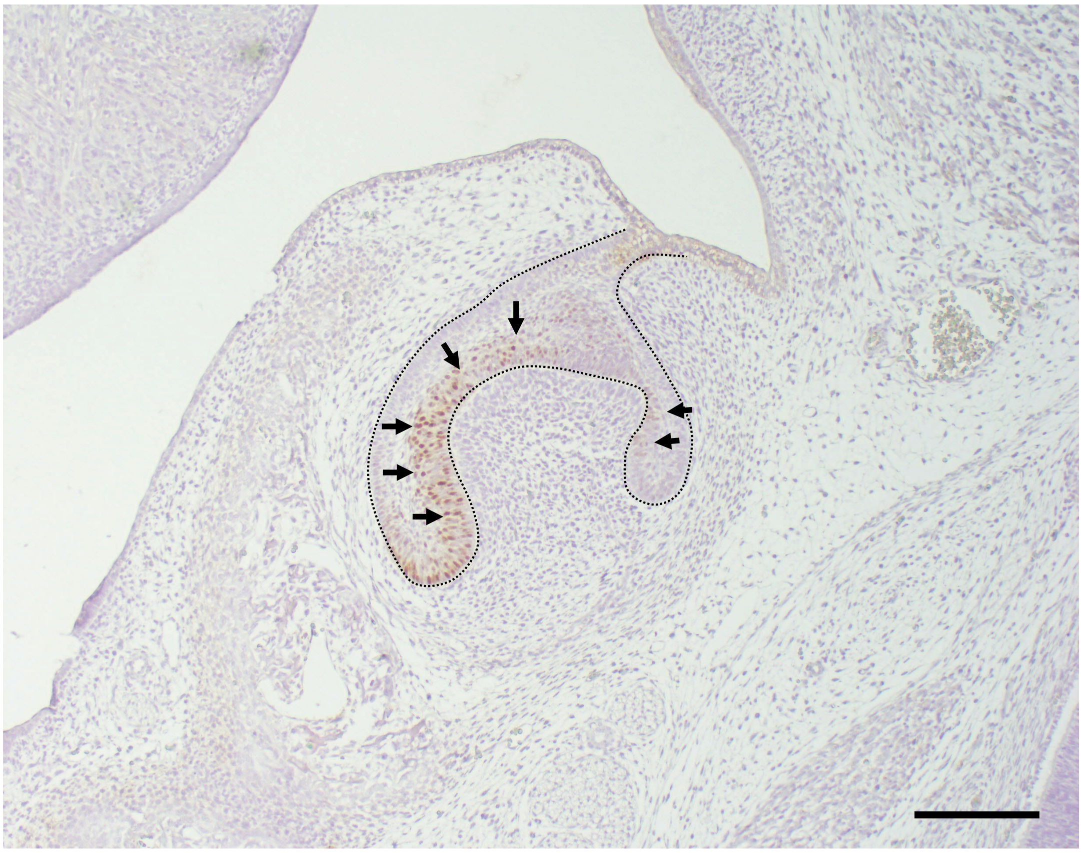

Immunohistochemical analysis of Epfn using anti-Epfn antibody (1:200) in developing mouse tooth germs of embryonic day 17.5.

Inner dental epithelial cells were positive for Epfn (arrows). Use of this antibody detected the limited expression of Epfn in inner dental epithelial cells with a very low non-specific signal. Dotted area indicates the enamel organ of the tooth germ. The antibody reaction was visualized with a peroxidase-polymer-based method using a Histofine Simple Stain MAX-PO kit (Nichirei) with diaminobenzidine (DAB, S Nichirei) as the chromogen, followed by counterstaining with hematoxylin. Immunohistochemistry was performed with three independent replicates. Bar indicates 200 μm

Supplemental Figure 2

A

```
                                                                          GATA
Human     ATTTGTTAAAAGTCTTTGCATAAGCCCCTTGTCAAGCCAAATGCTGTTTTCCTTTTAGTATCCAATTATC
Bovine    ATTTGTTAGAAATCTTTGCATAAACACTTTTCCA--GCCCACGCTGTTTTGCTTTTAATATCCAATTATC
Dog       ATTTGTTAGACATCTTTGCATAAACTCTTTTCCGAGGTCCACACTGTTTTCCTTCTAATATCCAATTATT
Mouse     ATTTGCTACATCTTCTTTTGCACTTTCTTTGTCAGAACCC-TGCTCATTTTCATCTACTATCTAGTTATC
Rat       ATTTGCTGCAACTTCTTTTGCAGATTCTTTGCCAGCACCT-TGCTCTTTTTCATCCATTATCTAAGTATC
                                    Sp       NF-y                  NF-y/VDER
Human     TGAAACTTAAGAAGAGTGTGCACCGCCCAATGGGTGTGTGTATGTGCTGCTTTGAACCTATAGTTGAGAT
Bovine    TAAAATTTAAGAAGAATGAGCACCGCCCCATGGGAGTGTGTGTGCTGCTCTATGATCCTATGGTTAAAAT
Dog       TAAAATTTAAGAGGAATGGGCACCGCCCAGTGAGGGTGTGTGTGTGTGACTTTGAGCCTATAGTTACAAA
Mouse     TGAAACTTTAGAGGAGTGGGCACCACCCCATGAGGGTATGTG---GCTGTTCTGATCCTGTGATTGAGAG
Rat       TGAAACTTTAGAGGAGTGGGCACCGCCCGATGAGGGTAGGTG---GCTGTTCTGATCCTATGATTGAGAA
                                                                          TATA
Human     CCAGAGAATTGGGAGTGACATCATCTGTAACAATAAAAGAGCCTCTCTTGGTAAGCAGAAGACCTATATA
Bovine    TCAGAGAATTGGGAGTGACGTCATCTGTAACAATAAAAAAGCTTCTC--AGT--GTGGAAGACTTATATA
Dog       CCAGAGAATTGGGAGTGACATCATCTATGACAATAGAAAAGCTTCTCTTGGTAAGTGGAAGACCTATATA
Mouse     CCAGAGAACCAGGAGTGACATCATCCTTAACAATAAAATA-CTCCTCTTGGTGAGCAAAAAGCCTGCATA
Rat       CCAGAGAACCAGGCATGACATCATCCTTCCCAATAAAATA-CTCCTCTTGGTGAGCAAAAGGCCTGCATA
                                        TSS
Human     T--AAAAGTCACCATTTAAGGGGTCTGCAGTCCAATTCATCAGTTGTCTTTAGTTTACTCA---------
Bovine    TATAAAAGTCAC-ATTGAAGGG-TCTACAGCTCAATTTATCAGCCTTCTCAGGTTTACTCAACTTTGAGA
Dog       T--AAAAGTCAC-ACTTGAGAG-TCTACAGTCAAATTCATCAGCTTTCACAAGTTTACTCAACTTCGAAA
Mouse     T--GAAACTCAGACTTGAAGAA--CTGCAGTCCAGTTCATCAGCTGTCT--GGTTTACTCCAGCTTACTA
Rat       T--GAAACTCAGGCTTGAAGAA--CTGCAGTCCAGTTCATCAGCTGTCT--GGCTTACTCCAGCTTAATA

Human     --GCATCAGCT------------ACTAACATACCTGAACGAAGA--------------------------
Bovine    AAGCATCAGCT------------GCTAATACATTTGAAAGAAGATTGTATCCTAAGACGT----------
Dog       AAGCATCAGCT------------GC---------------------------------------------
Mouse     CAGCATCAGTTTGTGCATCCCCGAAGGATCCCCTTTGAGAGTCATTGTATGGTAAGGAACCTCTCAACTG
Rat       CAGGGTCACT---------CCTGAAGGATCCTCTCTGAGAGTCATTGTATGGTAAGGAATCTCTCAATTG
```

B

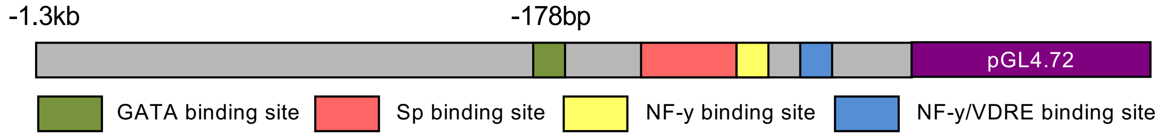

Sequence similarity and conservation of transcription factor responsive elements in PTH proximal promoter region among species.

(A) Alignment of conserved sequences in the PTH promoter region across human, bovine, dog, mouse, and rat genomes. (B) Schematic diagram of the luciferase reporter construct and GATA, Sp, NF-y, and VDRE regulatory sites of the 1.3-kb human PTH gene. Highlighted are GATA (green), Sp (pink), NF-y (yellow), and NF-y/Vitamin D response element (VDRE) (blue) binding sites.

Supplemental Figure 3

A DNA sequences of 5'-promoter (V1) region of human Epiprofin gene

```
-1000  TTCTGGTGAGAATTAGAAAGGAATGGTGGCAAGGGATAGCTCAATTCCTT
 -950  CCAGAAGTGTCCCTTCCCAGGGCCATTCAGGCGCCTGCCCATTCTTCAGA
 -900  ACCGGGTGCTCGGGGGCGAGGGTGTGCGTAGGCTGAGCATTTCCCGAAGG
 -850  ACAGGAGGGCGAGGAGAGGCGAATTTGAGAGGTGGATTTCTTAGGCACTG
 -800  GGGTTGCTACCTAAGGGGAGGGGCAGAGTGACAGCTGCTGGGACCTGGGA
 -750  CCAGAGTAGGGGGGAAGCAGAGACCGGTGGTGGGCAGAGGCTGGGAGAGG
 -700  GGAGGAGAGGAGGTGGTCACCCAGAAAGGTCAGAGAGGGATACCCCGGAA
 -650  GGGAGAGGGAAAAAAGGACGGGGAAGGGTGGTGGTGGAGAGAAGCGGCGC
 -600  GGGGTGCGGGGGGTGAGGCAGGGCAGGGGACGGGGGTCGGGGGTCGGGGG     GCMB
 -550  AATGGCGAAGGAGGGCAGCCAAGACAAATTTCACACGGCCTAGGCCTAGA
 -500  GGCGAACTGGAAACATACTTGGCGCTCAATCTTGTTTCCTTCTTTCCTCC
 -450  CAGTTCCGGGGCAGGGCTGTCCGTTCAGAGAGGGTCAAGGGCCATCGAGG
 -400  CAACCTAAGGGTTGCTGCCTAAGGGTGGCTCTGTCTCACCCCCAGGACCT
 -350  GCCCCCTTATCCATTCCCCCTGGGTCTTAGTCCTGCGTCCTCTGCTAGAC     GCMB
 -300  CGAGCTAGCTCCAGGGCAGTTTTCCACCCGGCGGGGCGGGTCTTAGAACC
 -250  TGGGAATCACCCCACCCGGGCCCCTCTCCCTGGGAGTGGAGGGGACCAGG
 -200  GAGGAGCGAGGGAGCGGGCGGCGCACGCCCTAGTTATCTTTCCTCCCCCA
 -150  CTCCCGCCCACCCCCTCCTCGCGGCCCTGGCTGGGCTCCGGTCCAGCCGA
 -100  GCCCTCAAGGGTTAAAGGCGGCCGCAGGTGAGGTGGGCGGGGCCGCGAGT
  -50  CCGGGGGAAAAAGCAGCGCTGGGGAGAGGATGAAGGCAGAGAGCGCGGGT
   +1  GAGTCACGGGCGGAGCTGGCCTTGTTCGCTCGCTGGCTCCTGCCCGCCCT
  +51  CCGTCCTCCGCCCTCTCGCCAGCGCTCACCTCCGCCGCCTGCCGCCTGCC
 +101  GCCAGCCGCCGGGTCTGGCTCGCCCTGGGCTCCTGCCCCTTACCGCTGGA
 +151  GAGCTCGCCGGCGCACAGGGCCTATGAGCGACCGTCAGTAGCGCACCAGC
 +201  CAGCCGTGCCCGGAGCCCGCCGCAGCCTCGAGgt
```

B DNA sequences of 5'-promoter (V2) region of human Epiprofin gene

```
-1000  GCTCATCCTGGGAGGGAGGTAGCATGTAAGTCTTGGGGTACTGGGTTCTG
 -950  GTGTGTGTAGAGGGGGTACAACTGCTCCTGTCCTCTGCCAAGGGCATTGG
 -900  GCTGTGGTCAAAAACAGGATGTCATCCCGTGAAAAGTCCCCAGGCCACTC
 -850  TCCTATGTGGGTCAGAGTTCACAGCTTCAGTGAGCTTTGGGAAACAAGTC     GCMB, AP1
 -800  TTACTCTTCTGTTTCATCTCCGTTCTCCTTCCTGGTCTTTTGTCCATCTC
 -750  TGCCCTGGGACATGTTTGTGTTAGCCAGTATACCTAAACTTACAACCACC
 -700  CTAAAATTAGGCTCATATAAATAGAGATCTGGGGGCTCCCCCTAAATTCA
 -650  CCTTCAGGCCCCTTCTCCCTTTTCCAGGCCACCTTCCTACCCTCTTCACC     NFAT
 -600  CTCCTGGAGTTCCAGGAGTCCTGAACTGTCTGTAGCAGTTCTTGGCTGGA
 -550  CTCTGCCTCTGGTCTTTCTGGTCTTAGGTGACCTCTTGTCCATCCTAAGC
 -500  TTCCCCACTCCATCCAAAGTCAGGGAAAAGGTCCTCCCTTCCCATTTGAC
 -450  AATGAGAGATCCCAGGTCCAAAAGATAGATTACACTGGGTCCTCTTGGGG
 -400  GATCCAAGTTGGGAAGTGAGTATGTAAAAAGAGGATGAAAAGGGAGCGAG
 -350  AGAAATTGTGGCTCAGAGAGGCCAACAACACTGACATGAAGCTCTGCAAG
 -300  GAGGAACTGACTTGAAGACGTGTGGTGTGTGTGTGTTTGTTGGGGGCATG
 -250  AGCCCTTCAGGTCTCAAAAATGGAACTTCTTTCTCTCTTACCAAGTCCAT
 -200  GGAGGAAAACTCAGGAAGAGGGGATGGAATTTGGAGAGTTCCAGCTATCC
 -150  CAGCTCTCCACCCTAGCAATGCTGTCATTTCCTCCATCCTGCAAGAACCA
 -100  CTTTGTCCCTCTCCCACTGCCCAGCCAACTCTGCGGAAGGAAGGAAGTGC
  -50  CCCCAGCCCCCACTCCCAGCTGAAATCCTCCTTTCCTGGCAGACCTCACG
   +1  GACTCAGATCTCACCTCCTACCACTCCCCTAGGAGAGCTGGGGGCCACTG
  +51  TTTCCTGGATTATCCTAAAAGCTTCTGAGGCCGTGAGGACTTGGCAGCAT
 +101  CCCTGCTCCCTCCTTCACCTCCCCCTTTGGCACTGCCTGTCACCTCCTTT
 +151  ATAAAGCCTGGCTCTTTTATCACCGCCACTTGGCCCTCACTGCCGCCGCC
 +201  AGCTCTGGGCTCCATGGACTGgt
```

DNA sequences of Epfn promoter variants

(A) Variant 1 exon 1 and (B) variant 2 exon 1 promoters. Sequences, presented in boxed regions, showed responsive elements for GCMB, AP1, and NFAT.

Supplemental Figure 4

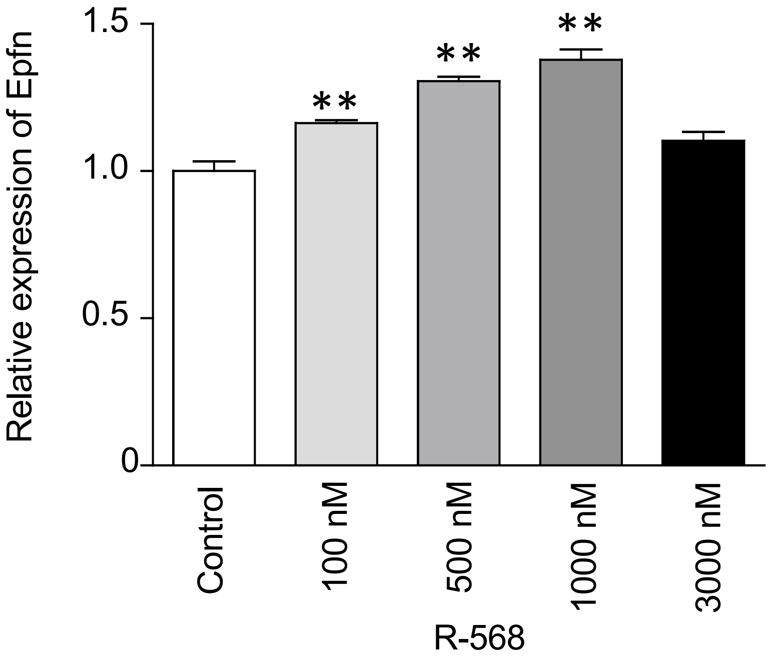

RT-PCR analysis of Epfn expression in PT-r cells cultured with R-568.

PT-r cells were maintained for 12 h in Ca2+-depleted medium with or without R-568. Epfn expression was induced by R-568 in a dose-dependent manner. **$P < 0.01$, as determined using the two-way ANOVA multiple comparisons test .

Supplemental Table 1

Expression profile of Epfn expression in sequence tag clone in NCBI UniGene

Organ	Human Hs.253603	Mouse Mm.156282
Bone	-	-
Bone marrow	-	-
Intestine	+	-
Kidney	-	-
Parathyroid gland	++	NL
Pituitary gland	-	NL
Thyroid gland	-	-

-: undetected, +: expressed, ++:strongly expressed, NL: not listed

Supplemental Table 2

Summary of PHPT patients age, blood test data, and histological diagnosis.

	Case 1	Case 2	Case 3	Case 9	Case 14	Case 15	Case 16	Case 17	Case 19	Case 20	Case 21	Case 23
**Age**	66	60	71	74	36	65	54	59	60	44	59	84
**Sex**	F	F	F	M	F	M	F	F	F	M	F	M
**1-84 PTH (pg/mL)**	57.6	104	84.5	83.8	57	182	80.1	88.7	93.9	38.6	329	159
**Ca (mg/dL)**	11.6	10.8	12.8	11.6	10	12	11.1	11.2	11.5	10.3	11.4	13.4
**IP (mg/dL)**	3.4	2.5	1.8	2.3	3	1.9	2.5	2.5	2.6	2.5	2.5	2.2
**ALP (U/L)**	422	296	291	195	182	339	293	369	434	181	544	346
**Weight (mg)**	1950	650	1475	550	650	2150	5900	600	1950	700	1600	1410
**Histological Diagnosis**	Adenoma	Adenoma	Adenoma	Adenoma	Adenoma	Adenoma	Adenoma	Adenoma	Adenoma	Adenoma	Adenoma	Adenoma

Supplemental Table 3

Primer list

RT-PCR and RT-qPCR

Mouse CaSR	5‘-CTTAGCCGTCTACTCCATTGCG -3’	5‘-TGAACAGTCTTTCTCCCTTCTTGG -3’	305
Mouse Epiprofin	5‘-TCAGCCTGCTTTGGGAGGATAC-3’	5‘-ATGCTTCTTCTTGCCCCCATCG-3’	313
Mouse PTH	5‘-CAGTTTGTGCATCCCCGAAGGAT-3’	5‘-TTTCACTGACAGCTCTCTTCCTCACG-3’	156
Mouse HPRT	5‘-GCGTCGTGATTAGCGATGATGA -3’	5‘-GTCAAGGGCATATCCAACAACA -3’	563
Rat CaSR	5‘-CTTAGCCGTCTACTCCATTGCG -3’	5‘-TGAACAGTCTTTCTCCCTTCTTGG -3’	305
Rat Epiprofin	5‘-TAACCTGCGAGGACCTGGAAAGTG -3’	5‘-ATGCTTCTTCTTGCCCCCATCG -3’	530
Rat PTH	5‘-TGTCTGCAAGCACCATGGCTAAG -3’	5‘-TTTCCCATCTGCCTGGGTAAGG -3’	220
Human Epiprofin	5‘-ACCTGGAAAGCGACAGTCC -3’	5‘-AACCACGATTCATAATGGTGTGA -3’	85
Human PTH	5‘-GAGTAGAATGGCTGCGTAAGAAG-3’	5‘-TTCATGGCTCTCAACCAAGAC-3’	134
Human CaSR	5‘-CCAACTTGACGCTGGGATACA-3’	5‘-CAGCAATCGTAGAGGGAATGTG-3’	156
Human Gcm2	5‘-CAAGGCACGGCTGAAACAG-3’	5‘-GCAGAATGACAGTTAGGGCAT-3’	51
Human CCND1	5‘-TTCGTGGCCTCTAAGATGAAGG-3’	5‘-GAGCAGCTCCATTTGCAGC-3’	108
Human/Mouse/Rat GAPDH	5‘-CCATCACCATCTTCCAGGAG -3’	5‘-GCATGGACTGTGGTCATGAG -3’	322

ChIP-qPCR

rPTH promoter -244F/-161R	5‘-ATCAGTATCATGGATTACGTCAGAT -3’	5‘-GTGCTGGCAAAGAATCTGCAA -3’	83
rPTH promoter -180F/-78R	5‘-TGCAGATTCTTTGCCAGCAC -3’	5‘-TCAGAACAGCCACCTACCCT -3’	102
rPTH +428F/+547R	5‘-GGCTGGCCTTGGGACAATAA-3’	5‘-CTCCTGAGAAGGCACAAAAA-3’	119
